# Supplementary material for: Assessment of frailty in patients with advanced chronic kidney disease, and the role of microvesicles: A single-center study
Source: PLoS One. 2025 Sep 30;20(9):e0332653. doi: 10.1371/journal.pone.0332653 (PMC12483209; doi:10.1371/journal.pone.0332653)
Supplement: Attachment — Submitted filename: Response To Reviewers.docx [file pone.0332653.s002.docx]

Response To Reviewers

We have made the following changes on our new manuscript file:

1. Changed the manuscript to meet the PLOS ONE's style requirements. Including page and line numbers, Titles changed, references updated. Also changed figures and tables in order to meet the correct style requirements.
2. We uploaded the figure files in “tiff” format, as requested.
3. Changed the “Competing Interests section” and included it in the cover letter.
4. We included a full ethics statement in the “Methods” section of the manuscript file. Including the full name of the ethics committee who approved our study. We have also uploaded the ethics committee document approval.
5. We reviewed and changed the reference list to ensure that it is complete and correct.
6. We have uploaded an anonymized data set (necessary to replicate our study findings), and this data has been uploaded as supporting information file.
7. This supporting information file contains all raw data required to replicate the results of our study.
