# Supplementary material for: Assessment of frailty in patients with advanced chronic kidney disease, and the role of microvesicles: A single-center study
Source: PLoS One. 2025 Sep 30;20(9):e0332653. doi: 10.1371/journal.pone.0332653 (PMC12483209; doi:10.1371/journal.pone.0332653)
Supplement: Attachment — Submitted filename: Response_to_Reviewers_auresp_2.docx [file pone.0332653.s003.docx]

# Response to Reviewers

We thank the reviewers for their thorough reading of our manuscript and the constructive comments, which have helped us improve the clarity and impact of our work. Below we provide a point-by-point response. All changes have been incorporated into the revised manuscript and highlighted accordingly.

**Reviewer #1**

**Comment 1:**

**The true novelty of your study is the possible role of microvesicles, but you haven’t emphasized that in either your Results or Discussion. As evidenced by your review of the existing literature, frailty among patients with CKD has a well-established body of evidence, but that’s what you emphasize in both your Results and Discussion. I recommend that you refocus your report and emphasize your findings regarding microvesicles. Otherwise, your study doesn’t tell us anything that we don’t already know.**

Response:

We appreciate this observation. In response, we have revised both the Results and Discussion sections to better emphasize the novel findings regarding circulating microvesicles (cMVs) in frail patients with advanced CKD. Specifically, we expanded our discussion of the implications of increased cMVs and CD142+ expression in frail patients:

“While frailty in CKD patients is well documented, our study provides novel insight by demonstrating a significant association between increased levels of circulating microvesicles and frailty. Frail patients had higher concentrations of both endothelial- and platelet-derived cMVs, and these cMVs also expressed more tissue factor (CD142), supporting a potential procoagulant phenotype.”… “These findings suggest that cMVs are not only markers of endothelial and platelet activation but may also reflect underlying pathophysiological mechanisms linking frailty with cardiovascular risk in CKD. Importantly, in our multivariable predictive model, total cMVs explained a meaningful proportion of frailty variability, independent of traditional factors such as age and T2DM. To our knowledge, this is the first study to establish such a link in this population. As such, cMVs may serve as promising biomarkers or therapeutic targets for frailty prevention and management in advanced CKD”.

**Comment 2 – Abstract:**

**The results you report here aren’t new except for the association between CMVs and frailty and that’s buried at the end of your results. The median followup isn’t really the point of your study. Report the data from the predictive model (Table 5). That’s far more meaningful than risk factors for frailty that was widely reported.**

Response:

We have revised the Abstract to include key findings from the predictive model (Table 5), specifically noting the contribution of T2DM, total cMVs, and hemoglobin to frailty prediction. Since the abstract should be limited to 300 words, we eliminated the results related to cohort follow-up, which are of lesser relevance.

“Results: The prevalence of frailty in patients with advanced CKD was 27% (CI 95% 17-37%). We found that risk factors for frailty were age (OR 1.06; CI 95% 1.01 to 1.11; p=0.027), type 2 diabetes mellitus (OR 5.77; CI 95% 1.76 to 18.9; p=0.004) and hemoglobin (g/dL) (OR 0.63; CI 95% 0.42 to 0.94; p=0.023). Total cMVs, platelet derived cMVs and endothelial derived cMVs were significantly higher in frail patients. In the predictive multivariable binary logistic regression model over frailty, three predictors explain 41% of the variability of frailty (Nagelkerke square R = 0.41; p <0.001) with the following contribution: T2DM (19%), total cMVs (14%) and hemoglobin (8%).”

**Comment 3 – Introduction (Line 101-102):**

**Being “highly prevalent” isn’t very meaningful. Report the prevalence and cite your source. For example, you cite a meta-analysis of prevalence by Veronese et al. in your Discussion. What was the prevalence they reported?**

Response:

We replaced "highly prevalent" with specific prevalence figures and cited the meta-analysis by Veronese et al. in the Introduction:

“Frailty affects approximately 10% of community-dwelling older adults, increasing with age [1].”

**Comment 4 – Introduction (Line 108):**

**The prevalence of frailty in ESRD is important but your focus is CKD. They’re not interchangeable. Can you report the prevalence in CKD as well, if only to provide a baseline for comparison with your own findings? Since your aim is to “analyze frailty” baseline data help readers contextualize your purpose.**

Response:

We added the information so that readers can contextualize the prevalence of frailty in the different stages of CKD:

“In fact, frailty is identifiable in approximately 35% of patients with CKD G3-5 (eGFR <60mL/min/1.73m2) [9], a percentage similar to that found in patients with advanced CKD (eGFR <20mL/min/1.73m2) [10] and patients undergoing hemodialysis [11,12] . Frailty is associated with higher risks of hospitalization and mortality [13-15].”

Taking into account this new information, we modified a sentence related to the prevalence in the discussion:

“The prevalence of frailty in patients with CKD is around 35%”

An we added new references:

9. Walker H, Carrero JJ, Sullivan MK, Field R, Lees JS, Hanlon P, Faucon AL, Fu EL, Beridze G, Jani BD, Gallacher K, Mark PB. Frailty in Adults with Chronic Kidney Disease and Validation of the Kidney Failure Risk Equation in Frailty Sub-Groups. Clin J Am Soc Nephrol. 2025 May 21. doi: 10.2215/CJN.0000000739. Epub ahead of print.

10.Kennard A, Richardson A, Rainsford S, Hamilton K, Glasgow N, Pumpa K, Douglas A, Talaulikar GS. Longitudinal frailty assessment in the prediction of survival among patients with advanced chronic kidney disease: a prospective observational single-centre cohort study. BMJ Open. 2024 Oct 10;14(10):e087189.

11. Zhang Z, Wang S, Xu Z, Sun Y, Zhou X, Zhou R, Li Q, Wang G. Frailty risk prediction models in maintenance hemodialysis patients: a systematic review and meta-analysis of studies from China. Ren Fail. 2025 Dec;47(1):2500663.

**Comment 5 – Methods (Line 146):**

**Can you please clarify what you mean by “conservative treatment?” Since it’s an exclusion criteria, readers should have a clear definition.**

Response:

We clarified that “conservative treatment” refers to patients with advanced CKD who chose not to initiate renal replacement therapy and were receiving only medical management without dialysis:

“Exclusion criteria included: patients undergoing renal replacement therapy or patients who decide not to initiate renal replacement therapy and were receiving only medical management without dialysis.”

**Comment 6 – Methods (Lines 150-151):**

**Please clarify how you measured “3 or more days of asthenia per week?”**

Response:

We clarified that this was assessed via patient self-report using a structured symptom questionnaire:

“3 or more days of asthenia per week (assessed via patient self-report using a structured symptom questionnaire)”

**Comment 7 – Results:**
**Because mortality and dialysis were your end-points, please report the number of patients for each outcome.**

Response:

We now explicitly state the number of patients who initiated dialysis (n = 23) and those who died (n = 4):

“23 patients (27.1%) began dialysis”.

“On the other hand, using univariate Cox proportional hazard models (only 4 deaths [4,1%] did not permit multivariate adjustment),”

**Comment 8 – Results (Lines 200-202):**

**Table 1 is not set up to support your statement regarding the prevalence of frailty in patients with type 2 DM and CVD. Table 1 compares frail and non-frail patients, not patients with or without type 2 DM and CVD.**

Response:

We have modified the sentence:

“Frailty was more prevalent in older patients, in patients with type 2 diabetes mellitus (T2DM) and in patients with cardiovascular disease as shown in Table 1.” has been modified to “Frail patients were older, with more type 2 diabetes mellitus (T2DM) and higher cardiovascular disease as shown in Table 1.”

**Comment 9 – Results (Lines 220-222):**

**You report using age, T2DM, hemoglobin and total cMVs for the regression model but haven’t reported any differences in cMVs. To justify using total cMVs, it would be helpful to readers for you report differences between frail and non-frail patients before you describe this analysis.**

Response:

We updated the Results (Table 1) to describe the differences in cMV concentrations between frail and non-frail patients prior to the regression model discussion. We added a sentence:

“Frail patients had higher concentrations of total cMVs.”

**Comment 10 – Results (Lines 242-244):**

**How were the variables for the hazard models chosen? Phosphorus wasn’t significant in the univariate or multivariate models.**

Response:

We clarified that variables for the multivariable Cox models were selected based on clinical relevance and statistical trends in univariate analysis:

Line 197: “Variables for the multivariable Cox models were selected based on clinical relevance and statistical trends in univariate analysis.”

**Comment 11 – Results (Lines 244-245):**

**You allude to mortality and dialysis here but haven’t reported the actual numbers. Mortality and dialysis data should be reported.**

Response:

As noted earlier, we now report the actual number of patients who initiated dialysis and those who died during follow-up.

**Comment 12 – Results (Line 275):**

**The correlations you report are statistically significant but cannot be described as “strong.” Please see statistical references such as:
Schober P, Boer C, Schwarte LA. Correlation Coefficients: Appropriate Use and Interpretation. Anesth Analg. 2018;126(5):1763-1768.**
[**https://doi.org/10.1213/ANE.0000000000002864**](https://doi.org/10.1213/ANE.0000000000002864)

Response:

We revised the wording to describe the correlations and decided to delete the word “strong”.

**Comment 13 – Line 286:**

**This is a minor point but I believe you have a comma instead of a decimal point for the Nagelkerke square value.**

Response:

Corrected. The value is now written as 0.41.

**Comment 14 – General (Discussion)**

**You haven’t made good use of your Discussion to highlight the novelty and contribution of your study. Focus on cMVs rather than well known outcomes such as prevalence and risk factors. For example, you discuss phosphorous at length but that wasn’t significant in either the univariate or multivariate model. It doesn’t contribute to the purpose of your study. Tell readers why your findings regarding cMVs are significant. What do they contribute to what we know about frailty?**

Response:

We restructured the Discussion to focus more prominently on the role of cMVs and their potential as biomarkers or therapeutic targets in frail patients with advanced CKD.

“While frailty in CKD patients is well documented, our study provides novel insight by demonstrating a significant association between increased levels of circulating microvesicles and frailty. Frail patients had higher concentrations of both endothelial- and platelet-derived cMVs, and these cMVs also expressed more tissue factor (CD142), supporting a potential procoagulant phenotype.”… “These findings suggest that cMVs are not only markers of endothelial and platelet activation but may also reflect underlying pathophysiological mechanisms linking frailty with cardiovascular risk in CKD. Importantly, in our multivariable predictive model, total cMVs explained a meaningful proportion of frailty variability, independent of traditional factors such as age and T2DM. To our knowledge, this is the first study to establish such a link in this population. As such, cMVs may serve as promising biomarkers or therapeutic targets for frailty prevention and management in advanced CKD.”

**Comment 15 – General (Line 340):**

**The correlations you found were statistically significant but they were not “strong.”**

Response:

We adjusted the wording in the Discussion and removed this word.

**Reviewer #2**

**Comment 1:**

**What exactly is the author's main purpose? To assess the prevalence of frailty in advanced CKD? Or the association between time to dialysis initiation and death?**

Response:

We revised the Abstract to clarify that our main objective is to evaluate frailty, and specially, its novel association with circulating microvesicles in advanced CKD:

“Our main objective was to evaluate frailty and its novel association with circulating microvesicles in advanced CKD.”

We also explain this in the Introduction:

“We aim to analyze frailty in patients with advanced CKD, and to determine the role of microvesicles in this population.”

Also to highlight the results of the main objective studied, we include in the abstract key findings from the predictive model (Table 5), specifically noting the contribution of T2DM, total cMVs, and hemoglobin to frailty prediction. Since the abstract should be limited to 300 words, we eliminated the results related to cohort follow-up, which are of lesser relevance.

“Results: The prevalence of frailty in patients with advanced CKD was 27% (CI 95% 17-37%). We found that risk factors for frailty were age (OR 1.06; CI 95% 1.01 to 1.11; p=0.027), type 2 diabetes mellitus (OR 5.77; CI 95% 1.76 to 18.9; p=0.004) and hemoglobin (g/dL) (OR 0.63; CI 95% 0.42 to 0.94; p=0.023). Total cMVs, platelet derived cMVs and endothelial derived cMVs were significantly higher in frail patients. In the predictive multivariable binary logistic regression model over frailty, three predictors explain 41% of the variability of frailty (Nagelkerke square R = 0.41; p <0.001) with the following contribution: T2DM (19%), total cMVs (14%) and hemoglobin (8%).”

**Comment 2:**

**Were the inclusion criteria simply age >18 and eGFR <20? frailty is extremely prevalent in the elderly population, and did the authors consider subgroup comparisons of older and middle-aged patients?**

Response:

We added a comment in Results and Discussion to clarify this:

Results:

“The age range of the sample was from 38 to 86 years.”

Discussion:

“The comparison of frailty by age is shown in Table 1 and was statistically significant in both the univariate and multivariate analyses (OR 1.06; 95% CI 1.01 to 1.11; p=0.027). In our sample, each additional year of age increased the risk of frailty by an average of 6%.”

**Comment 3 :**

**Statistical analysis, I would suggest the authors to describe in further detail, especially cox regression, as well as exploring the role of microvesicles.**

Response:

We expanded the “Materials and Methods” section to include more detail on the Cox regression model and statistical analysis of microvesicles:

“The start of dialysis (both haemodialysis and peritoneal dialysis) was evaluated using multivariable (controlled by T2DM, hemoglobin and phosphorus) Cox proportional hazards models.Variables for the multivariable Cox models were selected based on clinical relevance and statistical trends in univariate analysis. We use univariate Cox proportional hazard models for the calculation of the hazards ratios (HR) and P values of mortality (only five 4 deaths did not permit multivariate adjustment).”

“Pearson's correlation coefficient was used to evaluate correlations between cMVs and different parameters.”

**Comment 4** :

**Tables 1 and 2 could be combined**

Response:

We agree; we combine table 1 and 2.

**Comment 5:**

**Table 4 could be considered for replacement with Figure**

Response:

In the Table 4 we include 9 different correlations. Only three of them are statistically significant, and their presentation as a figure would not include a relevant amount of significant information. We would prefer to maintain the table and the figure containing just the relevant information (Fig 4). We agree that this is a way to give twice the same results, but we believe that the figure highlights the results included in the table. Nevertheles, if the reviewer thinks that the figure would be better than the table 4, we would make it without additional discussion.

**Comment 6**:

**Didn't the article use cox regression? Why do the authors mention in the limitation that “Also, the analysis using estimative binary logistic regression models can be adjusted for a limited number of predictor variables”.**

Response:

It should be clarified that the article uses multivariate binary logistic regression models for the analysis of frailty, and Cox regression models for the analysis of dialysis initiation and mortality. It is specified in Material and Methods:

“To define the risk factors associated with frailty, we conducted a univariate and multivariate analysis using estimative binary logistic regression models.”

“The start of dialysis (both haemodialysis and peritoneal dialysis) was evaluated, using multivariable (controlled by T2DM, hemoglobin and phosphorus) Cox proportional hazards models.Variables for the multivariable Cox models were selected based on clinical relevance and statistical trends in univariate analysis. We use univariate Cox proportional hazard models for the calculation of the hazards ratios (HR) and P values of mortality (only 4 deaths did not permit multivariate adjustment).”
